# Does It Matter What I Say? Using Language to Examine Reactions to Ostracism as It Occurs

**DOI:** 10.3389/fpsyg.2020.558069

**Published:** 2020-11-12

**Authors:** Fabian Klauke, Simone Kauffeld

**Affiliations:** Department for Work, Organizational, and Social Psychology, Institute of Psychology, Technische Universität Braunschweig, Braunschweig, Germany

**Keywords:** ostracism, LIWC, language, pronoun use, victim derogation, self-focus

## Abstract

Most of our knowledge related to how social exclusion affects those who ostracize and those who are being ostracized is based on questionnaires administered after the ostracism situation is over. In this research, we strived to further our understanding of the internal dynamics of an ostracism situation. We therefore examined individuals’ language—specifically, function words—as a behavior indicative of psychological processes and emergent states that can be unobtrusively recorded right in the situation. In online chats, 128 participants talked about a personal topic in groups of three. In the experimental group (*n* = 79), two conversation partners ignored every contribution by the third. We found that, compared to the control group, these targets of ostracism used language indicative of a self-focus and worsened mood, but not of social focus or positivity, although positivity was related to a writer’s likeability. Sources of ostracism used language suggesting that they were distancing themselves from the situation, and they further engaged in victim derogation. We discuss how our results highlight the severity and potential self-sustainability of ostracism.

## Introduction

Being ignored and excluded is an intensely painful experience (DeWall et al., 2010; Eisenberger, 2012; Ferris et al., 2019) and strongly motivates its targets to achieve re-affiliation (Williams, 2007). Ostracized individuals, thus, tend to try to connect with individual interaction partners (Maner et al., 2007), are motivated to work with others, join social clubs (Baumeister and Leary, 1995), or even join extreme groups (Hales and Williams, 2018).

In the long run, social isolation can have detrimental consequences for the individuals excluded, spanning physical and mental health issues such as depression, aggression, eating disorders, and higher mortality (Williams, 2001), but even seemingly trivial episodes of ostracism can be distressing (Nezlek et al., 2012; Hartgerink et al., 2015). Given the severity of the consequences ostracism has on the ostracized (henceforth called targets), imposing ostracism on others is perceived as a harsh violation of a general inclusion norm (Rudert and Greifeneder, 2016). Thus, it is strenuous for the individuals doing the ostracism (henceforth called sources) as well: They report emotional distress (Poulsen and Kashy, 2011; Legate et al., 2013), and find themselves in need for justification for their behavior (Nezlek et al., 2015).

Most previous research investigating ostracism has looked at participants’ judgments and behavior only after the ostracism situation has been concluded. Studies have tended to rely on questionnaires to ask participants how they felt while being excluded in an online ball-tossing game, but usually administer these questionnaires after the game itself is finished (Williams et al., 2000; Williams, 2009). This can be problematic since there is evidence that people’s recollection of their reactions to and ability to cope with negative events are biased (Todd et al., 2004), and this is particularly relevant for situations that conflict with one’s self-concept and are perceived as shameful or threatening to self-esteem, like ostracism (Williams, 2009; Wesselmann et al., 2016): Such shameful situations can prompt self-protective, defensive behavior (Barrett et al., 2002), and recollection of such events might be affected by various cognitive or motivational biases: self-serving biases, social desirability, or, more generally speaking, self- or other-deception. Thus, explicit measures about such socially sensitive topics are not considered to be always thorough or accurate (Barrett et al., 2002; Hofmann et al., 2005).

Taken together, while the temporal need threat model of ostracism suggests that the immediate, reflexive reaction to the situation differs from the more controlled, reflective reaction (Williams, 2009), only a few studies have directly measured truly reflexive reactions to ostracism. These studies have made use of physiological measures such as fMRI (Eisenberger et al., 2003), or specific mood dials to obtain continuous self-reports during experiments (Wesselmann et al., 2012). Aside from this handful of studies, what we currently know about ostracism may more accurately reflect whatever sense the involved individuals make of the situation afterward than what they experience in the situation itself. So what do sources of ostracism do to justify their actions *in the very situation*? What do targets *immediately* do to try and end the exclusion and achieve re-affiliation? How do they feel? Avoiding expensive and lab-locked technology, or biased and intrusive live self-reports, we look at individuals’ language when ostracism occurs. More specifically, we investigate individuals’ use of function words in ostracism situations. While content words—such as nouns and verbs—convey meaning, the use of function words such as pronouns or articles, has been linked to several psychological states and processes (Tackman et al., 2019; Tausczik and Pennebaker, 2010). For example, the use of personal pronouns can indicate where the speaker or writer puts his or her social focus (Zimmermann et al., 2013). To the best of our knowledge, the analysis of language has only been used to investigate reports of ostracism once (Klauke et al., 2020). In this study, it was found that when reporting ostracism, participants used language that indicated a stronger self-focus, lower connectedness, and higher complexity than when they were reporting an instance of social inclusion. However, like other previous studies on social exclusion, this study did not look at ostracism right as it was happening. Thus, in the present study, we aim to contribute to the existing literature in three ways: first, we plan to replicate previous findings on language use and ostracism, and extend it by applying language analysis to live ostracism situations, capturing a truly reflexive reaction to social exclusion. Second, we want to further our understanding of how and whether targets focus on themselves and others, and how they try to immediately achieve re-affiliation, by assessing their language. Third, we aim to examine the sources of ostracism: We want to know whether they engage in victim derogation, and what their language use could tell us about how they engage in dissonance reduction when actively ignoring someone.

## Targets and Sources of Ostracism

Social ostracism involves at least two parties: one party being ostracized, that is, ignored and excluded, and one party doing the exclusion, i.e., ignoring and excluding the former. Targets of ostracism are arguably more severely affected. They feel pain, suffer from negative affect, and their basic social needs—belongingness, self-esteem, control, and meaningful existence—are threatened (Baumeister and Leary, 1995; Eisenberger and Lieberman, 2004; Williams, 2009). Consequences of ostracism are often suggested to be more severe than other forms of (social) pain, as its targets are often not provided with a reason for their behavior (Sommer et al., 2001; Williams, 2009). This provokes rumination, causing the targets to introspect and come up with all kinds of self-related reasons for their treatment (Wesselmann et al., 2013a; Hales et al., 2016b). To re-fulfill their needs, targets first strive for reintegration, and, to that end, fine-tune their social perception. They become more attuned to social cues (Gardner et al., 2000; Pickett et al., 2004) and remember them better (Gardner et al., 2000). Furthermore, they get better at decoding these cues, e.g., distinguishing fake smiles from genuine smiles (Bernstein et al., 2008). On a behavioral level, this often leads to higher social servility (Williams, 2009): targets of ostracism cooperate more (Maier-Rigaud et al., 2010; Sheremeta et al., 2011), mimic potential interaction partners more (Lakin et al., 2008), express a greater desire to make friends (Maner et al., 2007), and are more willing to join even extreme groups (Hales and Williams, 2018). Taken together, this literature suggests that targets of ostracism are in a state of both self-focus and heightened attention to social cues, searching for connections with others.

As ostracism is so painful to those who experience it, people usually hesitate to harm others in such a way (Legate et al., 2013; Wesselmann et al., 2013b). In most situations, ostracism constitutes a violation of a general inclusion norm (Rudert and Greifeneder, 2016). Particularly the exclusion of likeable individuals is mentally straining to the sources of exclusion as well (Sommer and Yoon, 2013), and is considered immoral (Rudert et al., 2017). Ostracizing others, thus, does not only lead to feelings of guilt, shame, and even pain (Legate et al., 2013; Gooley et al., 2015; Nezlek et al., 2015), but also to the experience of cognitive dissonance (Festinger, 1957; Wirth and Wesselmann, 2018). When compensating the target for the inflicted pain (as in Wesselmann et al., 2013b) is not possible, research indicates several options for reducing dissonance. One way is victim derogation: perpetrators come up with reasons why committing such an offense is justified by devaluing the target (Festinger, 1957; Gawronski, 2012; Wesselmann et al., 2014). Another possibility is self-deception (von Hippel and Trivers, 2011): people can, for example, refuse to take full responsibility for their behavior (Schober and Glick, 2011) or play down the severity of one’s actions, decreasing their estimation of the pain they inflicted in others (Brock and Buss, 1962). Taken together, sources of ostracism are in need for justification of their actions.

In a first step, we thus expect that sources will try to reduce cognitive dissonance by devaluing the target of ostracism on basic, universal dimensions of social perceptions, i.e., warmth and competence (Fiske et al., 2002; Cuddy et al., 2008). Research so far has often hinted at the possibility that sources of ostracism engage in such victim derogation, but that assumption has, to our knowledge, not been systematically tested before (cf. Wirth and Wesselmann, 2018). Thus, we first want to establish whether:

*Hypothesis 1*: Sources will perceive targets as less warm (H1a) and less competent (H1b) than individuals not in an ostracism situation perceive each other.

The main goal of this study is to assess the effects of an ostracism situation on targets and sources as the situation unfolds. To that end, we employ an online chat paradigm where two participants were made confederates and ignored a third participant’s messages. There, we can record individuals’ language as immediate behavioral responses to ostracism.

### Language and Exclusion

Language is central to the coordination of groups and can signal several processes and emergent states (Van Swol and Kane, 2019). The words we use can be roughly differentiated into two categories: content words (e.g., nouns and verbs) and function words (e.g., pronouns and articles). Content words are words that carry a meaning which can, generally, be understood without further context or explanation (cf. Pennebaker, 2011). These meaning-bearing and relatively consciously chosen words are the traditional subject of content analyses and explore the ideas that people want to express (Boyd, 2017). Function words, however, have three advantages over content words that make them useful for the assessment of psychological states and traits: first, they are used independently of the topic that is communicated about. They are thus less reflective of the topic, but more of the author’s mindset (Pennebaker and King, 1999; Tausczik and Pennebaker, 2010). For example, suicidality of poets could be linked to how they used function words regardless of what their poems were about (Wiltsey Stirman and Pennebaker, 2001), and twitter users’ personality could be inferred by the way they tweet, regardless of what they tweeted about (Qiu et al., 2012). Second, they are used frequently, providing plenty of material for analysis: on average they make up more than half of the words used in a given text, although only making up a small percentage (about 1–2%) of the overall vocabulary (Pennebaker et al., 2015; Meier et al., 2018). Additionally, their use is almost automatic, and therefore, hard to control or manipulate (Chung and Pennebaker, 2007). Consequently, function words are minimally prone to motivational biases (Baumeister et al., 2007; Chung and Pennebaker, 2007; Cohen, 2012), making them particularly useful in assessing unpleasant or shameful memories and events like ostracism (Barrett et al., 2002). Function words have been found to signal how people relate to themselves and others (Zimmermann et al., 2013), and differ in reports of inclusion versus exclusion (Klauke et al., 2020). However, to the best of our knowledge, the language individuals use in ongoing situations of social exclusion has not been studied yet.

As laid out above, the state of targets of ostracism is one of disconnection, self-focus, and low status, while sources have to cope with cognitive dissonance and feelings of guilt. While targets want to achieve re-integration, sources need to reduce their cognitive dissonances, possibly via victim derogation and engagement in distancing behavior. These states and behaviors can find their representations in the language that individuals use, making language style—the use of pronouns, articles, and other function words—a useful and unobtrusive tool to study human interaction.

#### Use of Personal Pronouns

The way in which an individual refers to itself—via a collective “we” or an individualizing “I”—strongly relates to this person’s current state in relation to others (Zimmermann et al., 2013). The use of first-person singular pronouns (such as *I*, or *me*) seems to broadly relate to self-focus (Ireland and Mehl, 2014). It is connected to negative affect (Pennebaker and Lay, 2002; Tackman et al., 2019), depression (Edwards and Holtzman, 2017; Tackman et al., 2019), low status (Chung and Pennebaker, 2007; Kacewicz et al., 2014), and has been found to be used more in reports of social exclusion than in reports of social inclusion (Klauke et al., 2020). Furthermore, first-person singular pronouns are used less when individuals are distancing themselves from their behavior, or when they are deceiving themselves or others (Newman et al., 2003; Schober and Glick, 2011).

On the other hand, the use of first-person plural pronouns—like *we*—can reflect a collective identity (Brewer and Gardner, 1996; Sexton and Helmreich, 2000; Boals and Klein, 2005). Manipulating pronouns use leads participants to perceive relationships with friends as well as confederates on a task as closer and higher in quality when using “we” rather than “she and I” (Fitzsimons and Kay, 2004). Furthermore, the use of *we* has been shown to relate to a stronger perceived self-other-overlap in romantic relationships (Agnew et al., 1998). As another group of pronouns relevant to social interactions, third-person pronouns (e.g., *she*, *they*) have occasionally been linked to self-monitoring and general social awareness (Hoover et al., 1983; Ickes et al., 1986; Pennebaker et al., 2003).

Targets of ostracism suffer from a sense of lowered self-worth and disconnection, while also focusing on their social surroundings. Sources, on the other hand, try to distance themselves from the situation. We expect individuals’ pronoun use to reflect these states, and we examine the following hypothesis:

*Hypothesis 2*: While sources of ostracism will use fewer first-person singular pronouns (H2a), targets will use more first-person singular pronouns (H2b), fewer first-person plural pronouns (H2c), and more third-person pronouns (H2d), than individuals not in an ostracism situation.

#### Use of Articles

The use of articles (*a*, *the*) has often been found to be connected to more a formal or more distanced and abstract ways of writing or talking, as compared to a more narrative, personal style (Pennebaker and King, 1999; Heylighen and Dewaele, 2002). Individuals using a more article-heavy style are more likely to be of higher status: their use is positively related to both parental education and individual academic success, regardless of the academic subject (Pennebaker et al., 2014). Further, individuals low in article use tend to be more neurotic and agreeable (Pennebaker and King, 1999). Consequently, when reporting past experiences of social exclusion, people have been found to use fewer articles than when writing about inclusion (Klauke et al., 2020).

Summing up, articles are used more by individuals of higher status, when talking in a distanced, formal way, whereas agreeable, neurotic people use them less. Since we expect sources to try and distance themselves from the situation while targets are immediately put in a relatively low-status position, we hypothesize:

*Hypothesis 3*: Targets use fewer articles (H3a), while sources use more articles (H3b) than individuals not in an ostracism situation.

#### Use of Language to Increase Likeability

In essence, language is a tool to communicate. Its nature is therefore inherently social, and it is little surprising that some aspects of language, such as the use of positive emotionality, asking questions, or engaging in language mimicry, have been found to increase liking by others and foster relationship building. Since targets of ostracism feel disconnected and are in search for re-connection, these aspects are of particular relevance to this research.

##### Positive emotionality

The expression of positive emotion has been connected to (low-status) individuals seeking approval: Positive emotion words (such as *happy* and *nice*) were used more often by low status members in online forums (Reysen et al., 2010) and in e-mail negotiations (Belkin et al., 2013). Regardless of status, using positive emotion words increases the chance of reaching an agreement in online negotiations (Hine et al., 2009), and are used more by candidates before compared to after their election (Danescu-Niculescu-Mizil et al., 2013). Taken together, these results suggest the use of positive emotionality cues a warm self-image and increases an individual’s likeability and popularity.

##### Asking questions

Another conversational behavior linked to increased likeability is asking questions. In long-term relationships, people that draw out more information from their partners are rated as more likeable by their partners (Miller et al., 1983). Huang et al. (2017) found that asking questions signals responsiveness and increases liking in conversational partners, both in a natural environment and in an experimental setting when the number of questions asked was manipulated. There is also tentative evidence that low-status individuals ask more questions (Dino et al., 2009). This behavior makes sense, particularly in an ostracism context: if individuals want responses, a viable course of action would be to provoke those responses directly by asking.

##### Language style matching

Linguistic mimicry—mimicking the way one’s conversation partners are speaking—can be another way to affiliate with said partners, as the Communication Accommodation Theory (Giles et al., 1973) posits. Language divergence, on the other hand, can be used to express disaffiliation, to increase or emphasizes social distance, and usually leads to less liking (Gasiorek, 2016). A meta-analysis found that accommodation is consistently associated with positive evaluations of the communication, while divergence or non-accommodation is related to negative evaluations (Soliz and Giles, 2014). These findings have been extended to function words: mirroring an interaction partner’s linguistic style increases liking and can go as far as positively predict mutual romantic interest and relationship stability (Ireland et al., 2011), and particularly low-status individuals are evaluated as more empathetic when matching the language style of their conversation partners (Muir et al., 2016).

To sum up, the use of positive emotion words, questions, and the use of language style matching are used by individuals who are reaching out, trying to connect with others—while previously reviewed literature shows that ostracized individuals strive for connection and re-integration. We assume that targets of ostracism use language to achieve their goals and hypothesize:

*Hypothesis 4*: Targets use more positive emotion words (H4a), more question marks (H4b), and engage more strongly in language style matching (H4c) than the control group.

As laid out above, we assume that sources will distance themselves from the situation and therefore emphasize social distance. This distancing can be reflected in language divergence, so we postulate:

*Hypothesis 5*: Sources use more language divergence than the control group.

We assume that the use of positive emotionality, questions, and language style matching are viable tools to convey warmth, trustworthiness, and friendliness, so we examined:

*Hypothesis 6*: The use of positive emotionality (H6a), question marks (H6b), and Language Style Matching (H6c) increases warmth perceptions in conversational partners.

## Materials and Methods

### Participants

Participants were recruited via the psychological faculty’s mailing list and online social networks. They were grouped in teams of three for an online experiment involving a chat where they were all asked to talk about their favorite holiday destination. A total of *N* = 141 participants took part in our study for course credit, and/or to participate in a raffle to win 2 × €25. No-shows in the registered groups of three were substituted by confederates. This procedure had to be followed in five experimental groups where one source of ostracism had to be replaced each and for one participant in two control groups. The data of those confederates were excluded from analysis. To ensure that participants followed instructions, two judges checked all chat logs for sources’ replies to the targets. In two of the experimental groups, such replies were found, and these groups were excluded from the analysis.

The remaining *N* = 128 participants^1^ were, on average, 24.16 years old (*SD* = 5.36). A total of 80 identified as female, 47 as male, and one person did not answer. In the experimental group, 51 participants were instructed to be sources of ostracism, excluding another 28 participants as targets of ostracism. In the control group, which consisted of 49 participants, no participant received any further instructions.

### Procedure

Participants signed up to the experiment via an online calendar with their e-mail address, which was anonymous to other participants. When three participants signed up for any time slot, the group was randomly assigned to either the experimental condition or the control condition with a chance of 2:1. On the designated date, the group was sent an e-mail with a link to an online survey. This survey contained a short demographic questionnaire as well as login credentials and instructions for a subsequent group chat. In these instructions, all participants were asked to write about their favorite holiday destination, and to convince the others that their destination was the best. In the experimental condition, two of the three participants were individually instructed to ignore and exclude the third participant, and not to respond to any of their utterances.

After all participants were online, they were invited to a group chat and instructed by the investigator to start the discussion. After 15 min, the discussion was stopped by the investigator, who then sent a link to every participant in a private chat room. This link started the second part of their questionnaire, containing questions about their chatroom experience and their fellow participants.

This study’s procedure was reviewed and approved by the Ethics Committee of the Faculty of Life Sciences of the Technische Universität Braunschweig. The participants provided explicit informed consent to participate in this study both at the beginning of the experiment, and after the debriefing.

### Materials

#### Need Threat Questionnaire

Social need threat (Williams, 2009) was assessed via the German version of a semantic differential (Rudert and Greifeneder, 2016). It consists of one item for each basic need, judged on a nine-point scale [e.g., *rejected* (1) to *accepted* (9) for belongingness]. The total scale’s internal consistency in our study was α = 0.925. This scale served as a manipulation check.

#### Stereotype Content Model

The social perception of the other participants was assessed based on the stereotype content model, using four items for warmth and competence each (Fiske et al., 2002). Similar constructs have been used to assess the effects of victim derogation before (e.g., Hafer, 2000; Correia et al., 2012; Oldmeadow, 2018; Tepe et al., 2020). The items (e.g., *able* for competence, *friendly* for warmth) were translated to German and rated on a five-point scale. In our study, the internal consistency was α = 0.873 for the warmth scale and α = 0.848 for the competence scale. For our analyses, we used the mean of the scores by both other participants: Targets were rated by the two sources, participants in the control condition were rated by their two conversation partners. The rating of sources was not relevant to our analysis.

#### Language Analysis

The analysis of the linguistic style of participants’ chat protocols was conducted using the software LIWC 2015 (Pennebaker et al., 2015) with the German dictionary (Meier et al., 2018). This software counts words of any given text and classifies them into several linguistically and psychologically meaningful categories. It then reports each categories’ share of words of the overall word count. Before entering the texts in LIWC, corrections for typographical errors were made. Word recognition rate over all messages ranged from 78–94% per participant, with an average of 88%. Word recognition rate did not differ between conditions (all *p* > 0.394, *MD* < 0.606). Participants wrote an average of 184.91 Words (*SD* = 98.61). Word count differed by condition, *F* (2,125) = 14.12, *p* < 0.001, with targets using significantly less words (*M* = 110.64, *SD* = 62.18) than sources (*M* = 222.65, *SD* = 98.95) or the control group (*M* = 188.08, *SD* = 92.81).

#### Language Style Matching

To assess coordination of language style, we used the reciprocal LSM (rLSM) metrics for conversation-based individual rLSM and dyadic rLSM scores by Müller-Frommeyer et al. (2019). We used the individual scores for targets and participants in the control group, assessing their matching with both other conversation partners. For sources, we used the dyadic scores assessing the language style matching between sources and targets, as our hypotheses were not concerned with the language style matching of sources with each other.

#### Suspicion Check

We further gave participants the possibility to comment on the experiment in a text box. We checked their entries for notions of suspicion or improper adherence to the instructions. Two targets directly indicated suspecting a manipulation of which one specifically stated that he still felt awkward not being acknowledged. Two more participants mentioned they were wondering why they were excluded, and suspected experimental manipulation amongst other reasons. This insecurity about the reason for one’s treatment is typical for targets of ostracism, and is theorized to make them “consider a laundry list of bad things they have done or said” (Williams, 2009, p. 289). On the sources’ side, one participant indicated that the situation felt unnatural, another one commented that the instructions made it easier to strike up a conversation with foreigners (though not specifically referring to the exclusion instructions). A total of 12 sources indicated that they felt regret and/or that it was difficult for them not to reply to the target.

### Analysis

Group differences of warmth and competence and Language Style Matching were tested using an ANOVA and pairwise comparisons (one-sided) between the groups mentioned in the hypotheses. Data on word use frequency is, in essence, count data, and often noticeably non-normally distributed (Karlgren, 1999). It often follows rather a Poisson or binomial shape and is prone to zero-inflation, particularly in less frequently used categories. This holds also true for the data presented in this research, as examination of Q–Q-plots and Shapiro–Wilk statistics showed: Except for the *articles* category, all language category data deviated significantly from normal distribution in at least one condition, three categories (*we*, *other*, and *question marks*) across all conditions. *F-*tests and *t-*tests tend to handle non-normal zero-inflated data poorly, the use of rank-base tests is suggested instead (Šimkovic and Träuble, 2019). Thus, we examined group differences in language data using a Kruskal–Wallis test. Pairwise comparisons between the groups mentioned in the hypotheses were assessed using the Dunn’s test. Dependence between variables was assessed using the Kendall’s correlation.

Hypotheses 1 through 5 were tested between either sources or targets and the control group. Hypothesis 6—the hypotheses that positive emotionality, question marks, and language style matching positively affects warmth perceptions—was tested using data from the control group. Our reasoning for this is that sources of ostracism were instructed to ostracize targets, and we expected them to engage in victim derogation. Therefore, the relationship between target behavior and source judgment could be different in these participants. Consequently, we tested this hypothesis on the control group data, not affected by our manipulation.

For all directional hypotheses, *p-*values of direct comparisons or correlations are reported one-sided (cf. Cho and Abe, 2013; Lakens, 2016). Two-sided *p*-values are indicated as such.

## Results

To assess the quality of our paradigm, we checked whether the manipulation caused the assumed threat to the four basic social needs. We found that needs were substantially affected by condition, *F* (2,124) = 89.89, *p* < 0.001, and that the targets’ needs were less satisfied (*M* = 3.04, *SD* = 1.24) than those of participants in the control group [*M* = 6.91, *SD* = 1.68; *t*(76) = −10.86, *p* < 0.001], as a pairwise comparison showed. The need satisfaction of sources was even higher (*M* = 7.66, *SD* = 1.46) than in the control group, *t*(98) = 2.45, *p* = 0.016 (two-tailed). Investigating each need separately, we found that the targets had significantly lower need satisfaction on all four needs [all *b* ≤ −3.14, all *t*(75) ≤ −7.19, all *p* < 0.001, two-tailed], while sources scored slightly higher on all needs [*b* ≥ 0.71, *t*(75) ≥ 2.12, all *p* ≤ 0.036, two-tailed] but self-esteem [*t*(75) = 0.84, *p* = 0.403, two-tailed].

### Sources’ Perception of Targets

We expected the sources to rate targets lower on warmth (H1a) and competence (H1b) than participants in the control group would rate each other. We found that condition significantly affected ratings of warmth, *F*(2,125) = 8.14, *p* < 0.001, and competence, *F*(2,125) = 10.30, *p* < 0.001. Confirming our hypotheses, the targets were rated as less warm [*M* = 3.84, *SD* = 0.65; *t*(66) = −2.06, *p* = 0.021], and less competent [*M* = 3.19, *SD* = 0.63; *t*(66) = −4.23, *p* < 0.001], than participants in the control group (*M* = 4.14, *SD* = 0.54 for warmth, *M* = 3.84, *SD* = 0.58 for competence).

### Ostracism’s Effect on Language Use

Overall, we found various differences in the language that targets and sources of ostracism use when compared to the control group. Medians, quartiles, and the mean ranks for the assessed linguistic categories can be found in Table 1.

**TABLE 1:** Relative use of language categories (in percent of participants’ total word count) by condition.

	Target		Source		Control	
			
	Median (Q1–Q3)	Mean ranking	Median (Q1–Q3)	Mean ranking	Median (Q1–Q3)	Mean ranking
1st person singular	6.15 (4.53–7.38)	85.61	3.33 (2.57–5.21)	50.15	4.97 (3.12–6.14)	67.38
1st person plural	0.00 (0.00–0.00)	55.36	0.00 (0.00–0.40)	63.16	0.00 (0.00–0.54)	71.12
3rd person	0.74 (0.00–1.96)	67.80	0.65 (0.00–1.02)	66.51	0.52 (0.00–1.01)	60.52
Articles	5.55 (4.08–8.04)	50.02	7.39 (6.28–9.03)	72.97	7.48 (5.31–8.77)	63.96
Positive emotion	8.88 (6.23–12.76)	70.04	7.53 (6.57–9.96)	57.64	8.74 (7.32–10.56)	68.48
Question marks	2.43 (0.90–5.97)	75.13	2 (0.96–3.22)	66.40	1.41 (0.70–2.50)	56.45

*Median and quartiles represent the percentage of words used from each respective category. First and third quartiles appear in brackets under the medians. Mean rankings were obtained from the Dunn’s tests for comparisons between conditions.*

#### Use of Personal Pronouns

As expected, condition did significantly affect the use of first-person singular pronouns, *H*(2) = 17.00, *p* < 0.001. According to our hypotheses H2a and H2b, we found that targets used more first-person singular pronouns than the control group (*z* = 2.07, *p* = 0.019), while sources of ostracism used fewer such I-words (*z* = −2.32, *p* = 0.010).

While the overall effect of condition on the use of first-person plural pronouns was not significant, *H*(2) = 5.02, *p* = 0.081, planned comparisons revealed that targets were found to use “we” less frequently than the control group (*z* = −2.20, *p* = 0.014), confirming our hypothesis (H2c). However, no differences by condition could be found regarding the use of third-person pronouns; *H*(2) = 0.98, *p* = 0.613.

#### Use of Articles

The use of articles was affected by condition, *H*(2) = 6.94, *p* = 0.031. Pairwise comparisons revealed that neither targets (*z* = −1.59, *p* = 0.056) nor sources (*z* = 1.21, *p* = 0.112) significantly differed from the control group in their use of articles as predicted, but exploratory analysis showed that the targets used significantly fewer articles than the sources; *z* = −2.63, *p* = 0.009 (two-tailed), with the control group ranking in between (see Table 1).

#### Use of Likeable Language

We hypothesized that targets would use more positive emotion words. Differences in use of positive emotion words were not significant between conditions, though *H*(2) = 2.93, *p* = 0.231. The same was true for language style matching, which was not significantly affected by condition, *F*(2,125) = 1.89, *p* = 0.156. Although we did not find a significant overall difference between conditions regarding the use of question marks, *H*(2) = 4.75, *p* = 0.093, targets used more question marks than participants in the control group did (*z* = −2.13, *p* = 0.017), lending support to our prediction (H4b).

#### Language and Judgment of Warmth

In partial support of our hypothesis (H6a), we found that using more positive emotion words is related to others perceiving the writer as warm, τ = 0.235, *p* = 0.010. However, neither the use of questions (τ = −0.111, *p* = 0.138) nor language style matching *r*(47) = 0.215, *p* = 0.069 was related to warmth perceptions.

## Discussion

The present study investigated how language use is affected by ostracism as it occurs. We employed a chat paradigm where two participants were asked not to respond to a third participant. Sources readily followed orders to exclude the targets, and the manipulation effectively threatened the targets’ social needs. This paradigm then allowed us to investigate how linguistic style is affected by an ongoing ostracism situation. We found that both targets and sources of ostracism considerably differed from participants in a control group in their use of language.

As predicted, we found that targets of ostracism used more first-person singular pronouns but fewer first-person plural pronouns than participants in a control group with no ostracism. We did not find targets to use more third-person pronouns nor more “likeable” language: neither did they use more positive emotion words, nor did they match the linguistic style of sources more. However, targets did make greater use of question marks than the control group.

Sources, on the other hand, rated targets’ warmth and competence lower than participants in a control group rated each other. Sources also used significantly fewer first-person singular pronouns than individuals in the control group. Furthermore, we found that sources used more articles than targets.

### Targets’ Use of Language

Targets’ use of first-person pronouns fits well with the empirical results presented in our theory section, combining ostracism and language literature. By using more “I” and fewer “we”-pronouns, the targets’ language use reflects their inclusionary status. The increased use of I-talk indicates that their attentional focus shifts toward themselves. This shift has previously been linked to neuroticism (Yarkoni, 2010; Holtgraves, 2011; Qiu et al., 2012), which is characterized by a ruminative self-focus and negative thoughts (Teasdale and Green, 2004). Furthermore, the use of “I” has been positively linked to self-oriented impression management, i.e., Machiavellianism, but negatively related to a more other-oriented, accommodative, impression management (Ickes et al., 1986). Accordingly, this might be an explanation for why our ostracized participants in the reflexive stage of ostracism do not use more other-referencing pronouns (such as *they*, or *she*), which are thought to signal social awareness and self-monitoring (Hoover et al., 1983; Mehl and Pennebaker, 2003).

Further, we found no evidence for targets making an effort trying to come across as particularly friendly via the use of positive emotion words or engagement in language style matching. So why do targets of ostracism not use strategies readily (and presumably unintentionally) used by individuals before an election as well as low-status online community members seeking for approval (Dino et al., 2009; Danescu-Niculescu-Mizil et al., 2013)?

A possible interpretation lies in the temporal need-threat model of ostracism (Williams, 2009): targets of ostracism first enter a reflexive stage feeling pain and negative affect, and suffer from threatened social needs. They only begin to focus on re-fortifying these needs in the ensuing reflective stage. It is possible that targets are so stupefied by the unexpected exclusion that they only really react after a prolonged period of time. However, it was found that targets do adjust their behavior in compliance with group norms when threatened with exclusion (Kerr et al., 2009; Sheremeta et al., 2011), so in an ongoing ostracism situation, individuals have been found to try and achieve re-inclusion. Furthermore, we found that targets asked more questions than the control group, suggesting a prevailing interest in social interaction. We, therefore, offer a different, albeit speculative, explanation: individuals previously found to be using more positive emotion words were at least members of their respective communities. Targets of ostracism, on the other hand, are unsure about their status on a much more fundamental level and feel threatened—they might, thus, simply not consider it a good idea to present themselves as warm and open, particularly since high warmth perception tends to come at the expense of seeming low in competence, and therefore, vulnerable (Fiske et al., 2015). This would fit with a finding that ostracized individuals tend to become more disagreeable over time, and disagreeable individuals also tend to be ostracized more readily (Hales et al., 2016a).

Taken together, these findings hint at the gravity of ostracism, as the potential chain reaction of disagreeableness and ostracism could begin earlier than expected: targets’ focus shifts away from others to themselves. At the same time, they refrain from signaling agreeableness not only after a prolonged time but right in the moment of their exclusion. As ostracism is likely to be overdetected (Williams, 2009), this could not only increase the likelihood of ostracism persisting but could potentially turn trivial episodes of neglect into vicious cycles of ostracism. Our findings highlight the necessity of further investigation of the internal dynamics of an ostracism situation to substantiate these interpretations.

### Sources’ Behavior

Sources rated targets’ warmth and competence as lower than participants in a control group rated each other. As sources were instructed to ostracize the target, they had no *a priori* reason to assume lower warmth or competence in the targets. We interpret this as victim derogation: complying to unfairly treating others for no justified reason is known to cause cognitive dissonance (Festinger, 1957). A biased perception of others as more unfavorable is well suited to reduce such dissonance (Gawronski, 2012): cold and incompetent individuals or groups are readily met with contempt and rejection (Cuddy et al., 2008), and ostracizing more cold and incompetent people is regarded as comparably acceptable and less morally disgusting (Rudert et al., 2017). Thus, we argue that convincing oneself that one’s victims are cold and incompetent reduces cognitive dissonance and makes it more morally acceptable to exclude them.

Another way to reduce cognitive dissonance is to distance oneself from the behavior perceived as shameful or immoral (Schober and Glick, 2011; von Hippel and Trivers, 2011). Consequently, language use of the sources of ostracism hints at sources trying to distance themselves from their behavior: low amounts of self-references have been found to be associated with deceit both of others (Newman et al., 2003) and of the self (Schober and Glick, 2011). Although our finding that sources use more articles than targets was not predicted and should therefore be considered exploratory, it still lends tentative support to our hypothesis that sources try to distance themselves from the situation as article use is linked to a more factual, less narrative, and emotional linguistic style (Pennebaker and King, 1999; Heylighen and Dewaele, 2002).

It seems at odds with this interpretation that we did not find sources to use language divergence toward the target. We assume that this null finding could be due to our manipulation: sources were not ostracizing the target on their own volition but were complying with the experiment’s instructions. Therefore, they might be motivated to distance themselves from the situation but not from the target, as such behavior might further increase cognitive dissonance.

To summarize, by linking research on ostracism and language style, we were able to show how currently being a target or source of ostracism is represented in an individual’s language use. We found support for our hypothesis that targets focus on themselves right in the moment of the exclusion. Their language, however, did not indicate that they are particularly sensitive to their social surroundings or that they make any effort to come across as especially warm and friendly to achieve re-integration. We further were able to show that sources of ostracism devaluate their victims, and that they show linguistic signs of distancing themselves from the situation.

### Limitations and Future Directions

Although our study extends work on both language and ostracism research, our findings need to be contextualized within their limitations. Automated word count analysis is a coarse measure of language, ignorant of both context and content. Furthermore, the interpretation of language use as a signal for processes, e.g., the use of “I” as a sign of self-focus, is solely based on theoretical considerations, and therefore, a case of reverse inference. Thus, although such reverse inferences can have substantial predictive power (Hutzler, 2014), we can only assume that, e.g., it is actually self-focus that causes the use of first-person singular language. It is therefore particularly necessary to strictly differentiate between empirical findings and interpretations with regards to the current research.

Another limitation concerns the external validity of our paradigm. In general, the chat paradigm we employed is closer to ostracism seen in real life than in very abstract paradigms such as Cyberball (Williams et al., 2000). In our case, however, the sources were put in a forced compliance situation, asked to inflict (social) pain in another individual by excluding them, without any additional motivation to do so. This is important to keep in mind when interpreting our results on sources of ostracism: when having an actual motivation to exclude others, processes of reducing cognitive dissonance might be different. Nevertheless, our findings could lay the foundation for the analysis of inclusion and rejection in online communication such as group chats and online social networks or transcripts of face to face conversations. Future studies could back it up by analyzing transcripts of the language used in real-world ostracism situations.

Furthermore, our research left some questions unanswered. Contrary to our hypothesis, we neither found evidence for targets of ostracism showing linguistic signs of a focus on others nor using more positive emotionality, although, was the use of positive emotionality tied to the speaker being rated as warmer. Future research should investigate under which circumstances targets become more socially attuned and friendly when under the threat of ostracism, and how the words they use can help them reconnect and put an end to the exclusion. Understanding the internal dynamics of an ostracism situation and the actual behavior of both sources and targets can have important implications for helping targets reconnect and stop sources from causing psychological harm. Our research provides a first step toward such solutions.

### Conclusion

We found that both sources and targets of ostracism change their language in response to the different situations, signaling introspection and self-focus on the side of the targets, and distancing of the self from the situation on the sources’ side. Our findings suggests that the targets’ initial reaction to ostracism is not one of other-focus, and not one of attention to social cues, but a potentially detrimental self-focus, which has previously been associated with rumination, neuroticism, and depressive symptoms as well. Sources seem to avoid involvement in the situation. Together, this behavior could potentially turn a short episode of ostracism into a vicious circle.

## Data Availability Statement

The raw data supporting the conclusions of this article will be made available by the authors, without undue reservation, to any qualified researcher.

## Ethics Statement

The studies involving human participants were reviewed and approved by the Ethics Committee of the Faculty of Life Sciences of the Technische Universität Braunschweig. The participants provided their written informed consent to participate in this study.

## Author Contributions

FK conceived the original idea for this research, performed all analyses, interpreted the data, and drafted the manuscript. SK gave constructive feedback during the conceptualization phase of the study, helped in interpreting the results, and assisted with drafting the manuscript. Both authors approved the manuscript to be published.

## Conflict of Interest

The authors declare that the research was conducted in the absence of any commercial or financial relationships that could be construed as a potential conflict of interest.

## Footnotes

1 Following a power analysis, to achieve to achieve 80% power to discover medium effect sizes (*f* = 0.25) at *p* < 0.05 in an ANOVA, we aimed to recruit *N* = 159 participants. Due to time constraints and exclusions of participants not following our instructions, we ended up with *N* = 128. A power sensitivity analysis indicates that our tests thus yield a power of 80% to discover effect sizes down to *f* = 0.28.
